# Association between childhood trauma and Internet gaming disorder: a moderated mediation analysis with depression as a mediator and psychological resilience as a moderator

**DOI:** 10.1186/s12888-024-05863-4

**Published:** 2024-06-04

**Authors:** Qian Liu, Lijun Ouyang, Lejia Fan, Aijun Liao, Zongchang Li, Xiaogang Chen, Liu Yuan, Ying He

**Affiliations:** 1 Department of Psychiatry, National Center for Mental Disorders, National Clinical Research Center for Mental Disorders, The Second Xiangya Hospital of Central South University, Changsha, Hunan 410011 China; 2 Hunan Key Laboratory of Psychiatry and Mental Health, China National Technology Institute on Mental Disorders, Institute of Mental Health, Hunan Medical Center for Mental Health, Changsha, Hunan 410011 China; 3 The Second Xiangya Hospital of Central South University, No.139, Renmin Rd, Changsha, Hunan China

**Keywords:** Childhood trauma, Depression, Psychological resilience, Internet gaming disorder

## Abstract

**Background:**

The effect of childhood trauma on Internet gaming disorder remains unclear. In this study, we examined this association in Chinese students and explored the possible associated roles of psychological resilience and depression.

**Methods:**

In total, 8,579 students from Hunan Province, China, provided information regarding their sociodemographic factors, history of childhood trauma, any symptoms of depression, psychological resilience, and characteristics of Internet gaming disorder for this cross-sectional study. The impact of childhood trauma on Internet gaming disorder, as well as the extent to which it was mediated by depression and moderated by psychological resilience was evaluated.

**Results:**

The influence of childhood trauma on Internet gaming disorder was partially mediated by depression (*B* = 0.07, *95% CI* [0.04, 0.05], *p* < 0.001), with psychological resilience acting as a mitigating factor (*B* = -0.002, *95% CI* [13.74, 21.72], *p* < 0.001). Psychological resilience also moderated the association between childhood trauma and depression (*B* = − 0.003, *95% CI* [22.17, 28.10], *p* < 0.001). Our moderated mediation model elucidated psychosocial mechanisms, revealing the underlying link between childhood trauma and Internet gaming disorder. It also demonstrated the partial mediating role of depression and modulating role of psychological resilience among Chinese students.

**Conclusions:**

Education and interventions, along with effective social support, should be provided to enhance students’ psychological resilience and prevent childhood trauma and depression.

## Background

The incidence of Internet gaming disorder (IGD) among students is on the rise due to the rapid development of the Internet [1]. A meta-analysis of 113 epidemiological studies reported an average IGD prevalence rate of 2.47% in 31 different eastern and western countries, which was not influenced by year, region, or sample representativeness [2]. The prevalence rate of IGD was reported to be 5.5% among Chinese college students [3]. Over the last decade, research on IGD has increased significantly, highlighting its emergence as a public health concern as well as its contribution to adverse emotional states [4], including anxiety [5], depression [6], and suicidal tendencies [7]. Despite the existence of several promising treatment measures, effective and robust clinical treatments are scarce due to limited knowledge of IGD [8]. Therefore, recognizing the causes of IGD onset and perpetuation is imperative.

Numerous studies have found that interactions among biopsychosocial factors contribute to IGD [9]. The Interaction of Person–Affect–Cognition–Execution (I-PACE) model has emerged as the most significant theoretical framework in recent gaming studies [10]. According to this model, IGD results from interactions among predisposing factors, mediators, and moderators. The predisposition factor (e.g. childhood trauma) pertained to the fundamental characteristics of an individual. The mediating factor (e.g. depression) pertained to the responses to various situations. Coping styles (e.g. psychological resilience) were primarily defined as the moderating factor [10]. The relationships of these factors with IGD should be comprehensively understood to identify effective ways to reduce the incidence of IGD. However, the underlying mechanisms remain unclear and still need to be further explored.

Childhood trauma, a critical predisposing factor of IGD, encompasses various forms of psychological trauma, including neglect, abandonment, sexual abuse, physical abuse, and exposure to parents with severe mental illness [11]. The model shows that childhood trauma directly affects IGD, as recently observed in South Korea [9]. In addition, several studies have shown that students with mental health disorders, such as depression, are more likely to develop problematic gaming behaviour [12], and depression may mediate the association between childhood trauma and IGD [3].

While childhood trauma played a role in triggering IGD, it did not impact all individuals equally. In other words, the correlation patterns among childhood trauma, depression, and IGD were influenced by a moderator. Psychological resilience, defined as the capacity to efficiently adjust to, cope with, or overcome potential stress and dilemmas [13], could be a moderating factor in this process [14]. Increased psychological resilience indicates an individual’s ability to deal with adversity, adapt to new situations, and aid in their development [15]. Individuals exhibiting high levels of resilience have low susceptibility to the onset of mental health disorders [16, 17]. A previous study revealed a significant negative relationship between psychological resilience and the occurrence of child abuse or neglect [18]. A strong correlation was also observed between excessive weekly gaming, heightened perceived stress, and reduced psychological resilience [19].

To date, a previous study tested the mediating effect of depression in the relationships between childhood trauma and IGD in a small sample of Chinese college students [3]. However, this study failed to explore the moderating factors to buffer against the relationship between childhood trauma, depression, and IGD. Consequently, the present study addressed this gap in the literature. Based on the I-PACE model, we explored the relationship among these four variables within a broader Chinese student population through a multi-centre study with a larger sample size, which provided comprehensive prevention of and interventions for IGD. We developed a moderated mediation model to identify the relationship between childhood trauma, depression, psychological resilience, and IGD. The following hypotheses were tested: (1) childhood trauma, depression, and psychological resilience are associated with IGD; (2) depression mediates the impact of childhood trauma on IGD; and (3) psychological resilience moderates the relationships among these three variables.

## Methods

### Study design

**Fig. 1:**
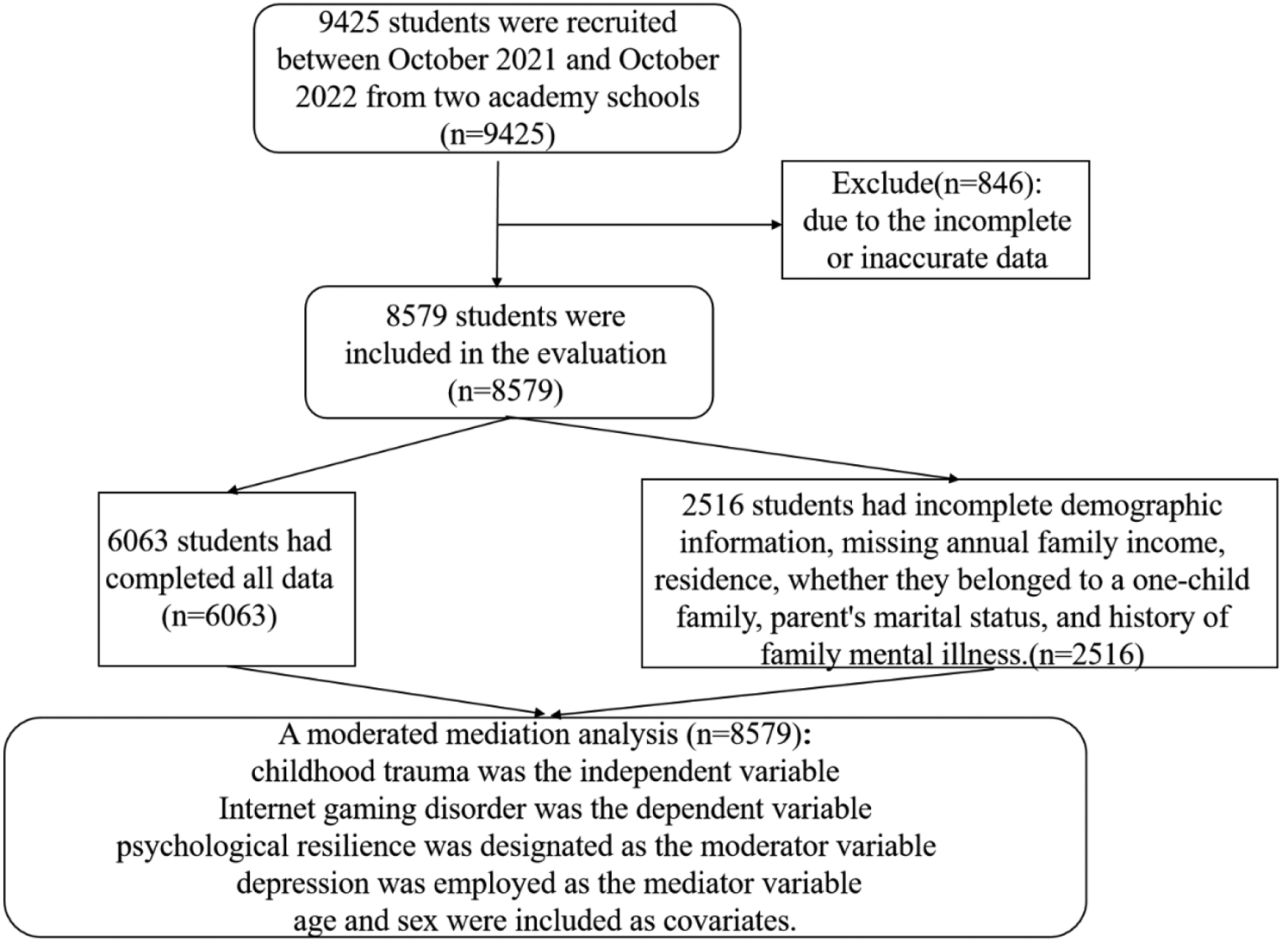
A flow chart of data analyzing. *Note* (1) childhood trauma = The Childhood Trauma Questionnaire Short Form score (2) Internet gaming disorder = The Internet Gaming Disorder Scale-Short Form score (3) depression = The Patient Health Questionnaire-9 score (4) psychological resilience = The Connor-Davidson Resilience Scale score

A cross-sectional online questionnaire survey was conducted in students between October 2021 and October 2022 in Changsha, Hunan, China (Fig. 1).

### Participants and procedures

We recruited 9,425 students aged 12–22 years from two academy schools that provided authorisation for data collection. Participants were selected through convenient sampling, meeting the inclusion criteria (i.e. students who were aged 12 years or above consented to participate and provide written informed consent and their guardians also gave written informed consent in this study), and completing the online questionnaire. Instructors presented information about the study, including its goals, survey structure, methodology, and confidentiality agreements, to the students before the commencement of the study. Data were collected through online psychological tests. Considering the confidentiality of participants, we uniformly blacked out all personal information and guaranteed complete anonymity after data collection to ensure data protection.

### Measures

#### Sociodemographic characteristics

We collected data on various sociodemographic characteristics of the students, including residence, age, sex, annual family income, whether they belonged to a one-child family, whether they were twins, smoking history, parents’ marital status, and a history of family mental illness.

#### The internet gaming disorder scale—short-form

According to the Diagnostic and Statistical Manual of Mental Disorders, Fifth Edition (DSM-5), diagnosing IGD requires a clinical interview, which is costly and time-consuming. Thus, the Internet Gaming Disorder Scale—Short-Form (IGDS9-SF), a brief psychometric tool based on the nine key criteria defining IGD suggested by the DSM-5, was developed by Pontes et al. [20] to assess IGD severity and its negative impact on gaming over 12 months. The response scale ranges from ‘never’ (score of 1) to ‘very often’ (score of 5), with total scores ranging from 9 to 45 [21]. According to the IGDS9-SF, the cutoff scores for IGD diagnosis are 32 and 36 for the Chinese and English versions, respectively [21, 22]. All participants self-scored the test. The IGDS9-SF presented with a high internal consistency in the Chinese Context (Cronbach’s alpha, 0.91) [22]. Our study demonstrated a strong internal consistency for the IGDS9-SF (Cronbach’s alpha, 0.940).

#### The connor–davidson resilience scale-10

The Connor–Davidson Resilience Scale-10 (CD-RISC10), a shortened Chinese version of the CD-RISC, is a 10-item self-assessment questionnaire assessing resilience or capacity to adapt to and cope with dilemmas [23, 24]. The Likert response scale ranges from 0 (‘never’) to 4 (‘all the time’). Higher scores indicate greater resilience. The Chinese version of the CD-RISC10 is a viable and reliable instrument for evaluating resilience in the Chinese population (Cronbach’s alpha, 0.92) [24]. The CD-RISC10 also demonstrated high internal consistency in this study (Cronbach’s alpha, 0.966).

#### The patient health questionnaire-9

Depressive symptoms were assessed by the Patient Health Questionnaire-9 (PHQ-9), a nine-item self-assessment questionnaire used to screen depression. The questionnaire uses a four-point Likert scale (0 = ‘never’ to 3 = ‘nearly every day’); higher scores indicate more severe depression. The PHQ-9 had a high internal consistency for Internet screening in depression among Chinese university students (Cronbach’s alpha, 0.8) [25]. In our study, PHQ-9 had strong internal reliability (Cronbach’s alpha, 0.902).

#### The childhood trauma questionnaire short form

The Childhood Trauma Questionnaire Short Form (CTQ-SF) is a 28-item self-reported questionnaire comprising 25 clinical and 3 validation items [26]. Respondents rate each item on a five-point Likert scale (1 = ‘never true’ to 5 = ‘very often true’). Higher scores indicate a greater exposure to abuse or neglect. The Chinese version of the CTQ-SF has demonstrated good reliability and validity for measuring childhood trauma in Chinese undergraduates (Cronbach’s alpha of the CTQ-SF total scale, 0.79) [27]. In our study, the CTQ-SF exhibited high internal consistency (Cronbach’s alpha of the CTQ-SF total scale, 0.797).

### Statistical analysis

Statistical analyses were performed using SPSS version 25.0 (IBM Corp., Armonk, NY, USA) and PROCESS macro 4.0. Descriptive statistics was performed to investigate the distributions of general demographic information. Pearson***’***s correlation tests were employed to explore the relationships between sex, age, childhood trauma, depression, psychological resilience, and IGD.

According to Hayes and Rockwood [28], the bootstrap methods of the PROCESS procedure in SPSS can analyse numerous types of data and run various statistical analyses for the mediation and moderation models based on bootstrapping (5000 bootstrap samples) using 95% confidence intervals (CI). The relationship was considered significantly mediated and moderated if a p-value of < 0.05 or the 95% CI of the interaction terms and indirect effects did not include zero. The CTQ-SF score served as the independent variable, while the IGDS9-SF score was the dependent variable. The depression score was employed as the mediator variable, the psychological resilience score was designated as the moderator variable, and age and sex were included as covariates.

The PROCESS Model 4 was employed to examine the mediating role of the depression score in the relationship between the CTQ-SF and IGDS9-SF scores. The moderated mediation analysis was examined using PROCESS Model 8. In addition, a simple slope analysis was used to test the correlation between the CTQ-SF score and IGD at different levels of the psychological resilience score. Similarly, a simple slope analysis was utilised to test the correlation between the CTQ-SF and the depression scores at different levels of the psychological resilience score.

### Ethics

The study procedures were performed according to the tenets of the Declaration of Helsinki. The Institutional Review Board of the Ethics Committee of the Second Xiangya Hospital of Central South University approved the study and consent was obtained from each participant. For participants under the age of 16 years, consent was obtained from their guardians. In addition, their guardians gave written informed consent for their respective minors to participate in the study.

## Results

### General demographic information

After excluding 846 responses due to incomplete or inaccurate data, 8,579 responses were included in the evaluation. The demographic characteristics of the 8,579 students are shown in Table 1; Fig. 1. Complete data were collected from 6,063 students, while 2,516 students had incomplete information on demographics, including missing information on annual family income, residence, whether they belonged to a one-child family, parent’s marital status, and history of family mental illness. Regarding the IGDS9-SF scores, most students scored < 32 (97.38%).

**Table 1:** Key demographic information and IGDS9-SF score of the samples (*N* = 8579)

Variable	*N*	%	NA	M + SD
**Sex**				
Male	3071	35.8		
Female	5508	64.2		
Age				16.89 ± 1.73
$<$15	437	5.1		
15–16	3183	37.1		
17–18	3327	38.8		
18–19	1494	17.4		
$\ge$20	138	1.6		
Yearly family income			2516	
$<$80,000	3262	53.8		
80,000-150,000	2077	34.3		
$>$150,000	724	11.9		
Residence			2516	
City	2743	45.2		
Villages	1537	25.4		
Countryside	1783	29.4		
Only one child			2516	
Yes	1504	24.8		
No	4559	75.2		
Twins				
Yes	560	6.5		
No	8019	93.5		
**Smoking history**				
Yes	186	2.2		
No	8393	97.8		
Parents’ marriage			2516	
Steady	5179	85.4		
Divorce	392	6.5		
Remarriage	249	4.1		
Single parent family	243	4.0		
History of family mental illness			2516	
Yes	284	4.7		
No	5779	95.3		
IGDS9-SF				14.53 ± 6.71
$<$32 scores	8354	97.38		
32–36 scores	137	1.6		
$\ge$36 scores	88	1.02		

*Note* IGDS9-SF = Internet Gaming Disorder Scale-Short Form

### Correlations among the variables

The IGDS9-SF scores were positively correlated with PHQ-9 scores and CTQ-SF scores (Table 2; *r* = 0.33 and *r* = 0.17, respectively; *p* < 0.01). However, the IGDS9-SF, PHQ-9, and CTQ-SF scores were negatively correlated with the CD-RISC10 scores (Table 2; *r* = − 0.23, *r* = − 0.46, and *r* = − 0.21, respectively; *p* < 0.01).

**Table 2:** Correlation among the scores of scales

	Gender	Age	Psychological resilience	IGD	Depression	Childhood trauma
Gender	1					
Age	0.19**	1				
Psychological Resilience	-0.02*	-0.11**	1			
IGD	-0.06**	-0.28**	-0.23**	1		
Depression	-0.02*	0.04**	-0.46**	0.33**	1	
Childhood Trauma	-0.25**	-0.12**	-0.21**	0.17**	0.21**	1
Mean	-	16.89	26.43	14.53	5.76	41.44
SD	-	1.73	8.65	6.71	5.07	11.34

*Note* (1) 1. Gender (1 = Male, 2 = Female) 2. Psychological Resilience = The Connor-Davidson Resilience Scale score 3. IGD = The Internet Gaming Disorder Scale-Short Form score 4. Depression = The Patient Health Questionnaire-9 score 5. Childhood Trauma = The Childhood Trauma Questionnaire Short Form score (2) ** *p* < 0.01; * *p* < 0.05

**Fig. 2:**
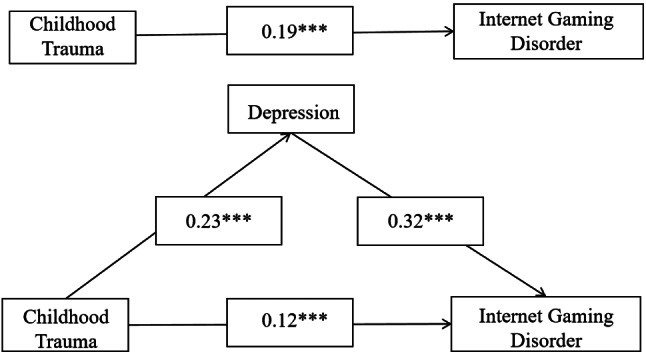
Mediated model predicting Internet gaming disorder based on the experience of childhood trauma mediated by depression. *Note* (1) 1. Childhood Trauma = The Childhood Trauma Questionnaire Short Form score 2. Depression = The Patient Health Questionnaire-9 score 3. Internet Gaming Disorder = The Internet Gaming Disorder Scale-Short Form score. (2) *** *P* < 0.001 ** *P* < 0.01 * *P* < 0.05

### Mediation model

As shown in Tables 3 and 4, and Fig. 2, childhood trauma significantly predicted IGD (*B* = 0.19, *t* = 21.46, *p* < 0.001), with the direct predictive effect of childhood trauma on IGD remaining statistically significant even after considering the influence of mediating variables (*B* = 0.12, *t* = 11.63, *95% CI* [0.06, 0.08], *p* < 0.001). The paths from childhood trauma to depression and from depression to IGD indicated significant associations (*B* = 0.07, *95% CI* [0.04, 0.05], *p* < 0.001; Tables 3 and 4, and Fig. 2). Statistical analysis revealed a substantial mediation effect of depression in the association between childhood trauma and IGD (Fig. 2), with the mediating effect accounting for 38.81% of the total effect (Table 4).

**Table 3:** Mediating role of depression on the associations between childhood trauma and IGD

	Dependent variable	Independent variable	β	t	*R*-sq	OR (95% CI)	F
Model 1	Depression	Childhood Trauma	0.23	18.39***	0.11	[0.10,0.13]	341.13***
Model 2	IGD	Childhood Trauma	0.19	21.46***	0.05	[0.09,0.11]	161.52***
Model 3	IGD	Childhood Trauma Depression	0.12 0.32	11.63*** 32.66***	0.21	[0.04,0.05]	554.33***

*Note* (1) 1. IGD = The Internet Gaming Disorder Scale-Short Form score

2. Depression = The Patient Health Questionnaire-9 score

3. Childhood Trauma = The Childhood Trauma Questionnaire Short Form score

(2) *** *P* < 0.001 ** *P* < 0.01 * *P* < 0.05

**Table 4:** Bootstrap results of direct and indirect effects and 95% confidence intervals between childhood trauma and IGD

	Effect size	SE	LLCI	ULCI	Relative effect value
Total effect	0.11	0.006	0.10	0.13	
Direct effect	0.07	0.006	0.06	0.08	61.19%
Indirect effect	0.04	0.003	0.04	0.05	38.81%

### Moderated mediation model

**Fig. 3:**
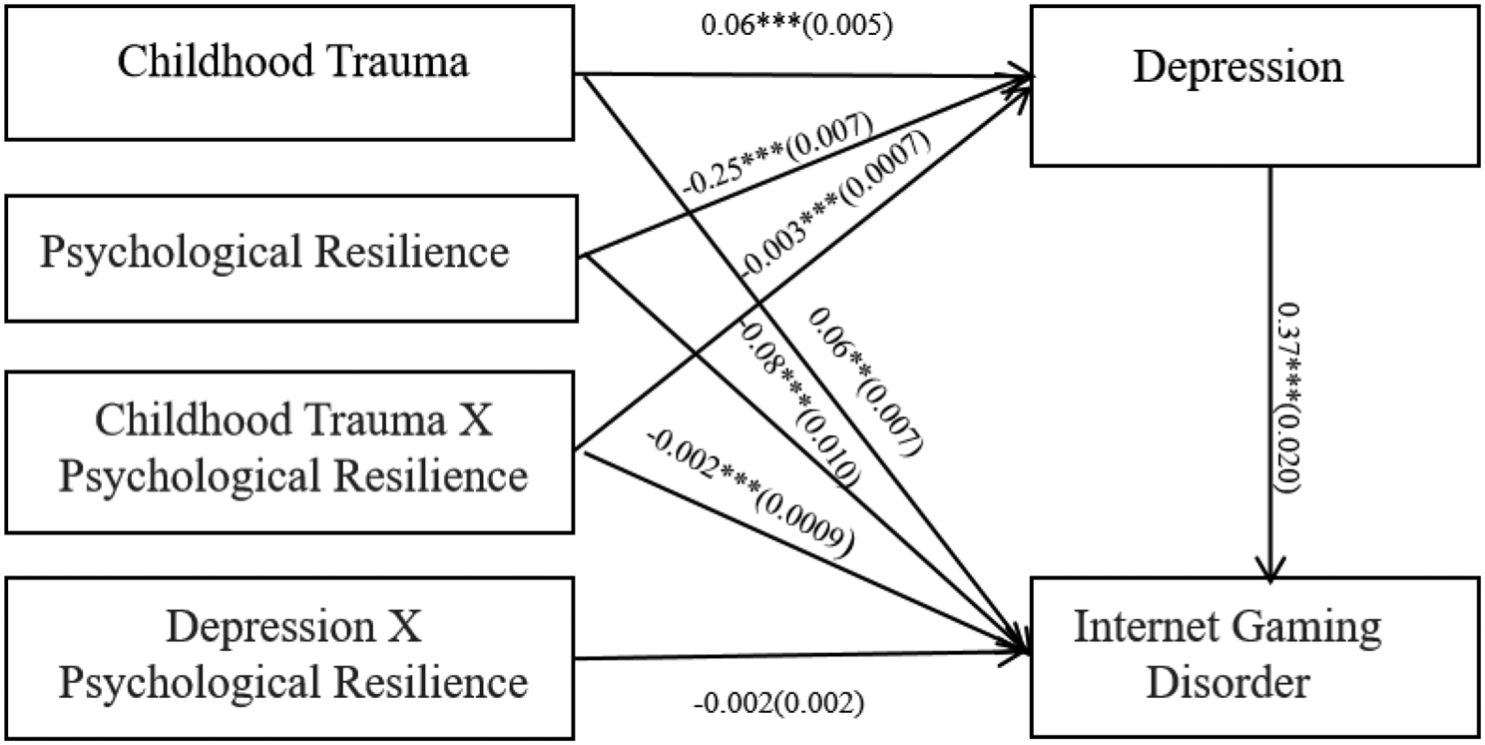
Model of the moderating role of psychological resilience on the indirect relationship between childhood trauma and Internet gaming disorder. *Note* (1) 1. Childhood Trauma = The Childhood Trauma Questionnaire Short Form score 2. Depression = The Patient Health Questionnaire-9 score 3. Internet Gaming Disorder = The Internet Gaming Disorder Scale-Short Form score 4. Psychological Resilience = The Connor-Davidson Resilience Scale score. (2) *** *P* < 0.001 ** *P* < 0.01 * *P* < 0.05

Tables 5 and 6; Fig. 3 showed the results of moderated mediation. The results showed that psychological resilience moderated the correlations between childhood trauma and IGD (*B* = − 0.002, *95% CI* [13.74, 21.72], *p* < 0.001) as well as between childhood trauma and depression (*B* = − 0.003, *95% CI* [22.17, 28.10], *p* < 0.001). However, psychological resilience did not moderate the correlation between depression and IGD.

**Table 5:** Summary of the moderated mediation model

	Dependent variable	Independent variable	β	t	*R*-sq	OR (95% CI)	F
Model 1	Depression	Childhood Trauma	0.06	14.30***	0.23	[22.17,28.10]	507.80***
		Psychological Resilience	-0.25	-42.55***			
		Childhood Trauma X Psychological Resilience	-0.003	-5.33***			
Model2	IGD	Childhood Trauma	0.06	10.54**	0.21	[13.74,21.72]	390.30***
		Depression	0.37	25.41***			
		Psychological Resilience	-0.08	-9.24***			
		Childhood Trauma X Psychological Resilience	-0.002	-2.73***			

*Note* (1) 1. Depression = The Patient Health Questionnaire-9 score

2. Childhood Trauma = The Childhood Trauma Questionnaire Short Form score

3. Psychological Resilience = The Connor-Davidson Resilience Scale score

4. IGD = The Internet Gaming Scale-Short Form score

(2) *** *P* < 0.001 ** *P* < 0.01 * *P* < 0.05

**Table 6:** Bootstrap results of the moderated mediating effects of resilience

Levels of resilience	Indirect effect	SE	OR (95% CI)
Low resilience	0.027	0.004	[0.020, 0.032]
Mean resilience	0.007	0.002	[0.003, 0.011]
High resilience	-0.013	0.004	[-0.021, -0.005]

**Fig. 4:**
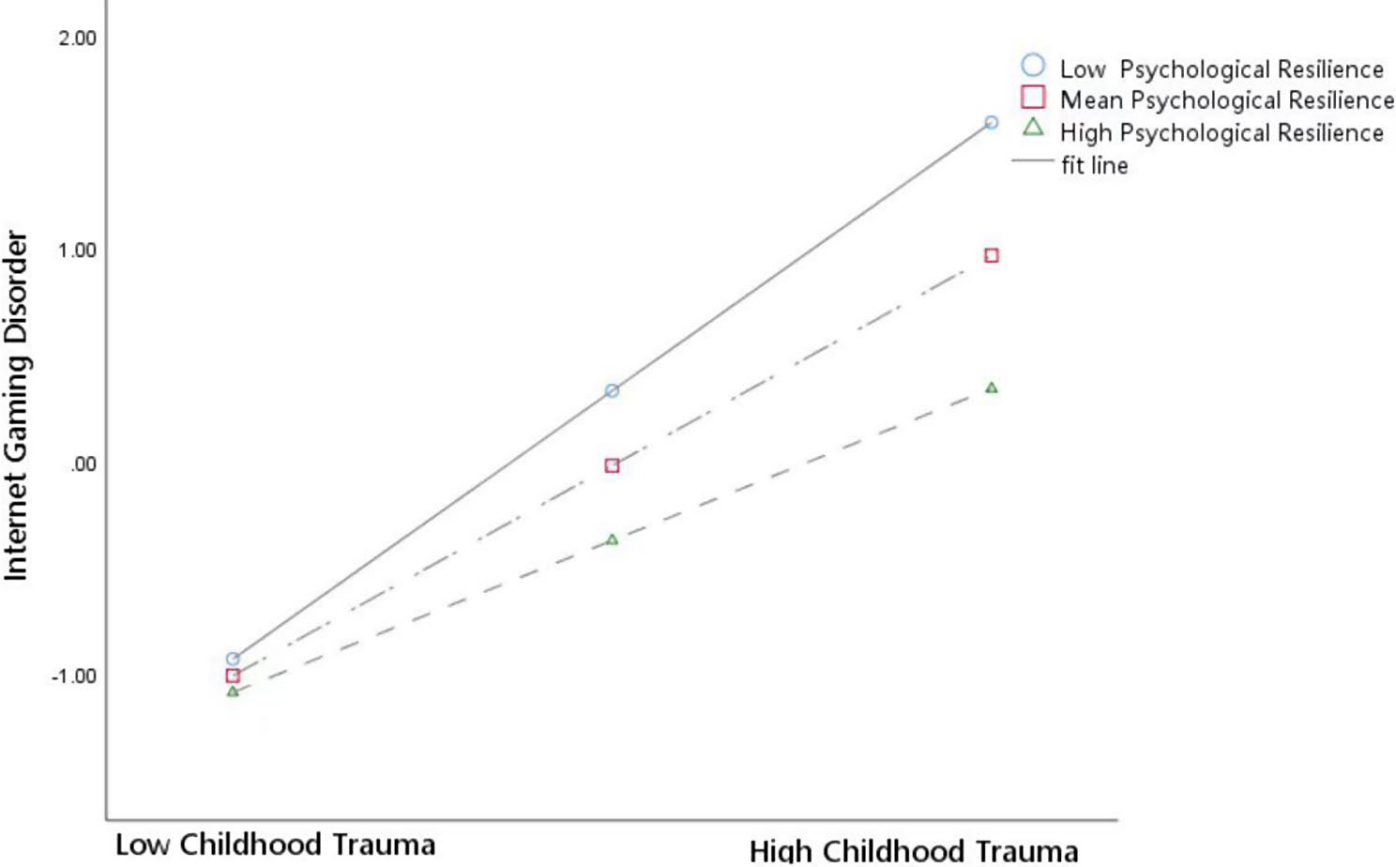
Moderating effect of resilience in the relationship between childhood trauma and Internet gaming disorder. *Note* (1) Childhood Trauma = The Childhood Trauma Questionnaire Short Form score (2) Internet Gaming Disorder = The Internet Gaming Disorder Scale-Short Form score (3) Psychological Resilience = The Connor-Davidson Resilience Scale score

In addition, we observed a stronger positive correlation between childhood trauma and IGD in students with low psychological resilience (*t* = 11.09, *95% CI* [0.08, 0.12], *p* < 0.001) than in students with high resilience (*t* = 7.72, *95% CI* [0.04, 0.07], *p* < 0.001) (Fig. 4). When exposed to the same levels of childhood trauma, students with poor resilience had higher IGD scores.

**Fig. 5:**
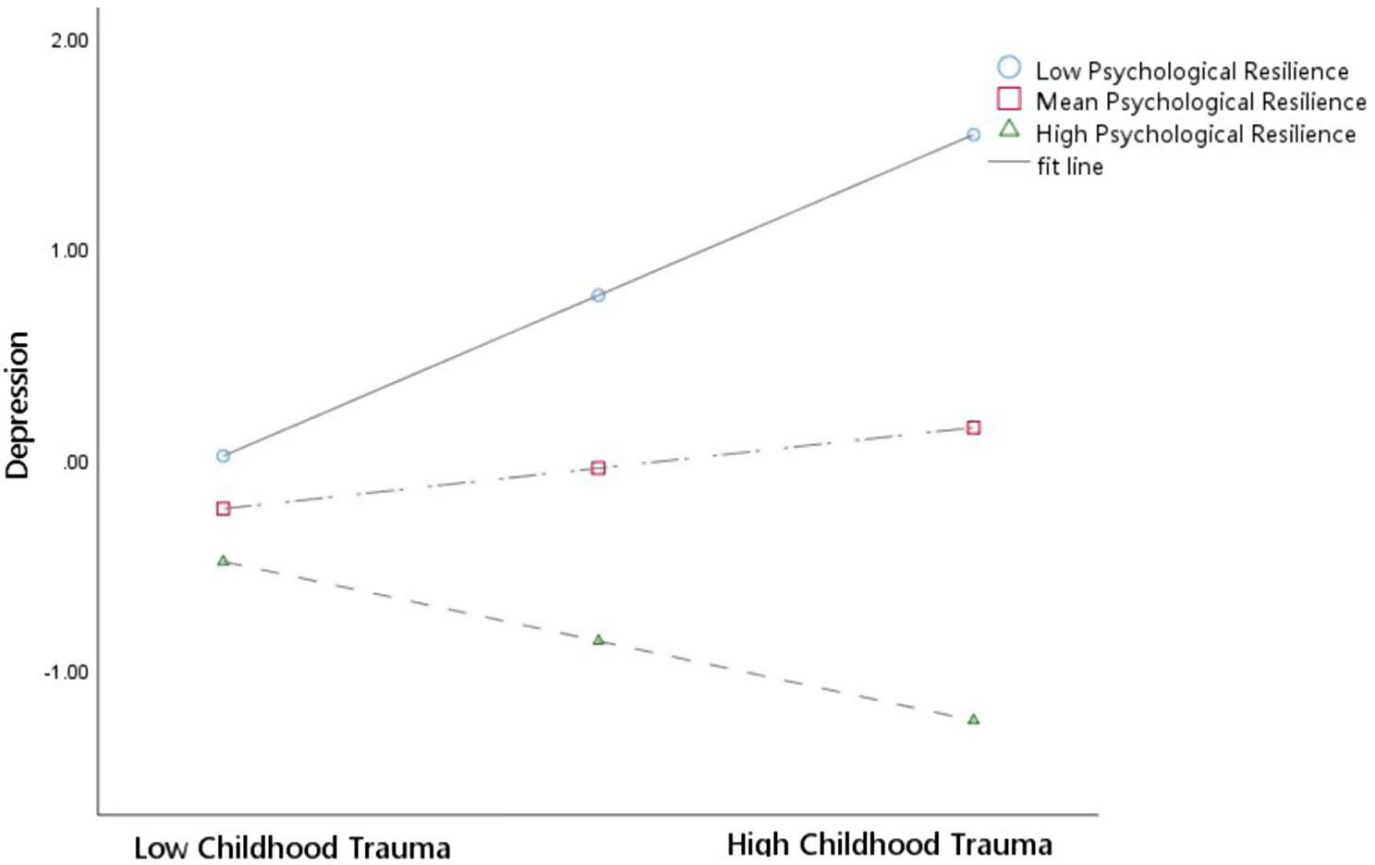
Moderating effects of resilience in the relationship between childhood trauma and depression. *Note* (1) Childhood Trauma = The Childhood Trauma Questionnaire Short Form score (2) Depression = The Patient Health Questionnaire-9 score (3) Psychological Resilience = The Connor-Davidson Resilience Scale score

Similarly, we assessed the moderating impact of psychological resilience on the association between childhood trauma and depression (Fig. 5). For students with low psychological resilience, childhood trauma significantly positively influenced depression (simple slope = 0.06, *t* = 9.24, *p* < 0.001). Conversely, childhood trauma significantly negatively predicted depression in individuals with high psychological resilience (simple slope = − 0.03, *t* = − 5.60, *p* < 0.001).

## Discussion

We formulated a moderated mediation model based on the I-PACE model regarding IGD, with childhood trauma as the predisposing factor, depression as the mediating factor, and psychological resilience as the moderating factor. Over 8,000 students participated in our cross-sectional survey. Our findings indicated the following: (1) childhood trauma, depression, and psychological resilience were important factors of IGD; (2) depression acted as a partial mediator in the impact of childhood trauma on IGD; and (3) psychological resilience was a moderator between childhood trauma and depression, as well as between childhood trauma and IGD. This study enhanced our comprehension of the association between childhood trauma and IGD.

Our study demonstrated that childhood trauma was a potential predictor of IGD, with the childhood trauma score significantly correlating with IGD severity. Similarly, numerous surveys have consistently demonstrated that childhood trauma was associated with mental health issues within the Chinese context [29, 30]. It is prevalent in Chinese families, which may be related to the Chinese family nurturing style. Many parents have perceived physical punishment and strict parenting as manifestations of care and affection, as evidenced by the proverb, ‘beating and scolding represent love’ [31]. Studies have shown that individuals who experienced childhood trauma tended to feel less parent affection, exhibited poorer self-esteem and self-worth, and were more prone to developing IGD as a coping mechanism for loneliness and being loved [32, 33]. In addition, numerous studies have revealed that childhood trauma impaired neuroplasticity and neurogenesis in brain circuits that regulated emotion and motivation, which was the biological basis for susceptibility to addictive behaviours [34, 35]. Therefore, it is crucial to promote popular science information. For example, parents should provide positive parenting and stable parent-child relationships, to prevent childhood trauma.

Our mediated model illustrated that depression significantly mediated the association between childhood trauma and IGD among students. This finding aligned with previous research indicating that traumatic experiences, directly and indirectly impact IGD through depression [3]. A potential explanation is that childhood trauma impaired the students’ emotional self-regulation, which made them choose a maladaptive strategy to relieve negative emotions, such as resorting to online games [36, 37]. In addition, this mediating role of depression was associated with cognitive distortions. Based on Beck’s cognitive model of depression [38], these students may generalise the negative effects of the traumatic event to other aspects of their lives, leading to avoidance of the real world and escape-seeking behaviors, such as addiction.

Our study also revealed that psychological resilience moderated the association between childhood trauma and IGD. Students with elevated psychological resilience demonstrated better coping mechanisms in the face of childhood trauma, which reduced the likelihood of developing negative behaviours [39]. Conversely, compromised executive control of students with childhood trauma and low psychological resilience made them fail to distinguish between actual reality and virtual reality, which enhanced the likelihood of gaming addiction [40]. Moreover, a previous study showed that building psychological resilience could reduce the occurrence of IGD by setting limits and enhancing self-control skills [41]. These perspectives, coupled with the current results, suggest that a well-rounded facilitated psychological resilience is the foundation for adolescents to enhance their self-control skills and reality ability in the future, which subsequently helps them restrain their internet gaming behaviour and are less prone to IGD. When childhood trauma is irreversible, psychological resilience should be used as an intervention to cultivate and strengthen psychological resilience.

Our findings indicated that psychological resilience served as a buffer against the detrimental effects of childhood trauma on depression. Among students with lower mental resilience, a significant positive association was observed between childhood trauma and depression. In contrast, students with higher resilience exhibited a significant negative association between childhood trauma and depression. The possible explanation of this result is that students with higher psychological resilience demonstrated enhanced emotional regulation skills and a reduced likelihood of experiencing heightened negative emotional arousal [42]. In addition, this regulatory effect of mental resilience may be related to neurotransmitters in the brain. A prior study found that students with elevated psychological resilience levels may possess more sensitive dopamine receptors, experience positive emotions, and employ adaptive coping strategies [43]. Hence, psychological resilience plays an important role in the relationship between childhood trauma and depression. However, we cannot clearly determine how psychological resilience buffers the harmful effects of childhood trauma on depression. As such, more social and biological factors can be further incorporated to explore the mechanisms of psychological resilience in the future. Regardless, our research results underscore the importance of psychological resilience and provide relevant research evidence for the development of courses related to psychological resilience.

However, psychological resilience did not act as the only moderator in the relationship between depression and IGD. Psychological resilience was characterised by the positive emotional and behavioural adaptation to adversity [44]. Its counteractive impact was more pronounced in the early stages of stressful events [45]. The protective influence of psychological resilience may be constrained when depression occurs. Hence, psychological resilience should be considered as an early intervention to mitigate the adverse effects of childhood trauma.

In conclusion, our study proposes comprehensive preventive and intervention measures to reduce the occurrence of IGD. Firstly, changing parenting styles is an extremely critical prerequisite to avoid childhood trauma in Chinese families. Secondly, for childhood trauma, early intervention, such as mindfulness therapy and cognitive behavioural therapy should be actively implemented to prevent depression. Finally, our findings highlight the importance of psychological resilience. Early interventions, such as psychoeducation and group cognitive-behavioural therapy, should be implemented to strengthen psychological resilience, including recognising and changing negative thinking patterns, effective emotional regulation, and active problem-solving skills development.

Our study had some limitations. First, convenient sampling might have introduced selection bias and the exclusion of participants with incomplete data might have skewed the results. Our future efforts will focus on conducting random sampling, collecting larger and more diverse samples, and minimising the manual handling of samples to enhance the generalisability of our findings across different populations. Second, the cross-sectional design prevented the exploration of causality. To address this limitation, we plan to conduct longitudinal studies in the future. Specifically, prospective studies that systematically evaluate the fluctuations in childhood trauma, depression, psychological resilience, and IGD will provide valuable insights. Third, a discrepancy existed in the sociodemographic data completion rates. Future studies should collect complete sociodemographic data, which will allow us to control for the interference caused by the variations in these factors. Fourth, we did not consider potential cultural or contextual differences that could affect the validity and reliability of these measures. Future studies should consider these factors. Finally, we used the total childhood trauma score as the observed variable, without delving into the various types of childhood trauma. Future studies should examine the association between different types of childhood trauma and IGD.

## Conclusion

The influence of childhood trauma on IGD was found to be partially mediated by depression, and the correlation between childhood trauma and both depression and IGD was moderated by psychological resilience. Based on our research, we recommend that this model is implemented as a potential framework for the prevention of and interventions for IGD. Implementing education and interventions to bolster students’ psychological resilience and prevent depression, coupled with providing effective social support from families, schools, and society, is crucial for preventing childhood trauma.

## Data Availability

All data generated or analysed during this study are included in this published article.
